# Supplementary material for: Causal interplay between lactose intolerance and gut microbiota: a combined bidirectional Mendelian randomization and in vivo validation study
Source: Front Nutr. 2026 Jun 1;13:1803337. doi: 10.3389/fnut.2026.1803337 (PMC13265576; doi:10.3389/fnut.2026.1803337)
Supplement: Supplementary file 1 [file Data_Sheet_1.zip › supplementary materials/Forward/leave one out cross validation/ebi-a-GCST90027528.finngen_R12_E4_LACTONAS.pdf]

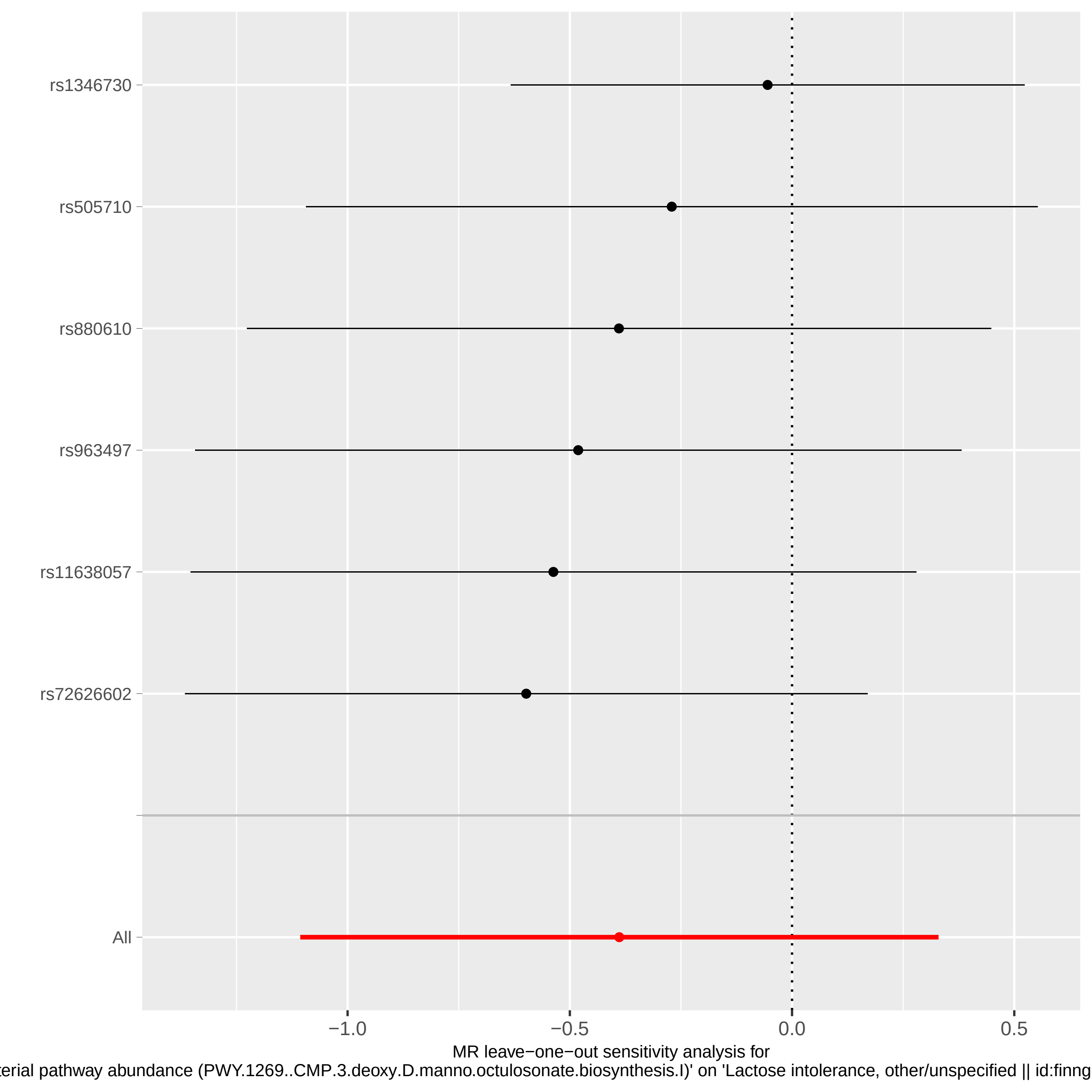
rs1346730
rs505710
rs880610
rs963497
rs11638057
rs72626602
All
−1.0
−0.5
0.0
0.5
MR leave−one−out sensitivity analysis for
terial pathway abundance (PWY.1269..CMP.3.deoxy.D.manno.octulosonate.biosynthesis.I)' on 'Lactose intolerance, other/unspecified || id:finng
